# Cervical Cancer Prevention: New Frontiers of Diagnostic Strategies

**DOI:** 10.1155/2015/250917

**Published:** 2015-06-10

**Authors:** Massimo Origoni, Walter Prendiville, Evangelos Paraskevaidis

**Affiliations:** ^1^Department of Obstetrics & Gynecology, School of Medicine, Vita-Salute San Raffaele University, 20132 Milan, Italy; ^2^UPMC Beacon Hospital, Dublin, Ireland; ^3^Division of Obstetrics & Gynecology, School of Medicine, University of Ioannina, Epiros, Greece

Invasive cervical cancer still represents one of the major issues of preventive oncology either in developed countries or in developing countries, accounting for the fourth leading cause of cancer related deaths in women worldwide and the second leading cause of deaths in women in developing countries [1]. It is the greatest cause of cancer death in women in sub saharan Africa, even outstripping breast cancer. Since the introduction of population-based organized cytological screening programs, a dramatic decrease of incidence of cervical cancer has been obtained in many western countries; despite this reassuring result, the overall performance of cervical cytological screening, the Pap test, is still far from being optimal [2]. Because Human Papillomavirus (HPV) is the recognized necessary cause for the development of cervical cancer, the present research is aimed at the investigation of new approaches towards its prevention and is particularly focused upon several biomolecular patterns and detection tools of the virological contamination of the lower female genital tract. The identification of high-risk viral strains DNA (HPV 16 and 18) is now worldwide recognized as more effective than cervical cytology in several settings: primary screening, triage of atypical cytology, and follow-up after treatment [3]. In this model, the traditional approach of looking for the early stages of the cervical neoplastic disease has nowadays shifted to the biological interpretation of the effects of HPV persistent infection on cervical epithelia and thus to the identification of “at-risk” individuals or groups rather than affected patients. The concept of risk stratification for cervical cancer is the final result of a cultural revolution in the field of cervical cancer natural history knowledge and isolation of progression risk factors. Molecular techniques are better than cervical cytology with respect to diagnostic sensitivity and reproducibility to detect cervical intraepithelial neoplasia grade 2 (CIN2) or cervical intraepithelial neoplasia grade 3 (CIN3)—the high-grade lesion precursors of invasive cervical cancer. According to data from four European randomized trials comparing HPV-based cervical cancer screening with cytology-based cervical cancer screening, HPV-based screening resulted in a 60–70% reduction in invasive cervical cancer incidence compared with cytology-based screening. The decrease in incidence of invasive cervical cancer with HPV testing was not significant within 2.5 years of enrolment, but the effect became decisive with longer follow-up [4]. Besides these results that have significantly contributed to the shift to a molecular screening strategy, a large amount of research works has been performed and is still ongoing in the field of identifying additional and novel aspects of the HPV causal effect of determining the neoplastic transformation of cervical tissues: genotyping, E6/E7 oncoproteins overexpression, high-risk HPV mRNA determination, novel progression biomarkers such as p16^INK4a^ and Ki67, DNA methylation markers, and, last but not least, the future role of colposcopy in the biomolecular era. In this special issue, readers will find articles that focus on these promising aspects of investigation, providing interesting results that could potentially open new windows of knowledge and new options of interventions in the still unfinished battle towards cervical cancer that started with Papanicolaou.

*Massimo Origoni* *Walter Prendiville* *Evangelos Paraskevaidis*
